# Anatomical Variations of the Sciatic Nerve Exit from the Pelvis and Its Relationship with the Piriformis Muscle: A Cadaveric Study

**DOI:** 10.3390/neurolint14040072

**Published:** 2022-10-31

**Authors:** Juan Pablo Reynoso, Manuel De Jesus Encarnacion, Renat Nurmukhametov, Dmitry Melchenko, Ibrahim E. Efe, Evgeniy Goncharov, Angel Alfonso Taveras, Issael Jesus Ramirez Pena, Nicola Montemurro

**Affiliations:** 1 Department of Anatomy, Autonomous University of Santo Domingo, Santo Domingo 10014, Dominican Republic; 2 Department of Neurosurgery, Russian People’s Friendship University, 121359 Moscow, Russia; 3 Department of Spine, Clinical Hospital of the Russian Academy of Sciences, 121359 Moscow, Russia; 4 Department of Pathological Anatomy, Central Clinical Hospital of the Russian Academy of Sciences, National Research Medical University, 121359 Moscow, Russia; 5 Department of Neurosurgery, Charité–Universitätsmedizin Berlin, Corporate Member of Freie Universität Berlin, Humboldt-Universität zu Berlin, and Berlin Institute of Health, 10117 Berlin, Germany; 6 Traumatology and Orthopedics, Clinical Hospital of the Russian Academy of Sciences, 121359 Moscow, Russia; 7 Morphological Department, Autonomous University of Santo Domingo, Santo Domingo 10014, Dominican Republic; 8 Department of Oncology, Royal Melbourne Hospital, Melbourne, VI 3005, Australia; 9 Department of Neurosurgery, Azienda Ospedaliera Universitaria Pisana (AOUP), University of Pisa, 56100 Pisa, Italy

**Keywords:** piriformis muscle, sciatic nerve, anatomical variations, anatomy, neuroanatomy, neurosurgery

## Abstract

Background: The sciatic nerve (SN) is the widest nerve of the human body that exits the pelvis through the greater sciatic foramen, usually below the piriformis muscle (PM), and descends between the greater trochanter of the femur and ischial tuberosity of the pelvis to the knee. The aim of this paper is to examine and identify the SN variations in relation to the PM, its prevalence, pattern, and course. Methods: A prospective-descriptive cross-sectional study was carried out to determine the frequency of anatomical variations in the exit of the SN in relation with the PM in 20 anatomical bodies (corpses) of both genders, in equal numbers. Results: The dissection of 40 SNs in corpses of both sexes in equal numbers showed that the SN exited inferior to the PM in 37 lower limbs (92.5%); between the fascicles of the PM and inferior to the PM in two lower limbs (5%); and in one thigh, between the fascicles of the PM and superior to the PM (2.5%). Our study reported that the SN divides in its terminal branches more commonly in the proximal part of the popliteal fossa in 55% of cases, in the gluteal region in 35% of cases, and in the middle third of the thigh in 10% of cases. Conclusions: Anatomical variations of the SN in relation to the PM are challenging for the diagnostic and therapeutic procedure in many clinical and surgical cases. Rapid recognition of the SN changes makes surgical approaches more accurate and effective. Our study confirmed that the SN exits the pelvis most commonly below the PM, although some anatomical variations may occur.

## 1. Introduction

The sciatic nerve (SN) is the longest and widest nerve of the human body, formed from the L4-S3 ventral roots and normally exiting the pelvis via a single trunk, through the great sciatic foramen usually below the piriformis muscle (PM). The SN courses in the posterior thigh compartment and divides itself into the tibial nerve and the common peroneal trunk at the popliteal fossa. The variations adopted by the SN at its emergence from the pelvic cavity after its formation by the branches of the sacral plexus, resulting basically in the relationship of the nerve with the PM, which are very well established [1]. Anatomical variations of the SN in relation to the PM have been described for many years and the possible implications these variations might play in certain pathologies. The correct identification of the anatomical variations in the population are important to identify patients for correct medical care, as the SN block is routinely used for anesthesia and analgesia during foot and ankle surgery, as also for minimally invasive techniques to provide neurostimulation therapies for chronic pain [2,3,4,5]. Ultrasound can be utilized to localize a specific nerve, view neighboring soft tissue anatomy, and plan a needle trajectory.

The SN is the terminal branch of the sacral plexus, formed in the pelvis from the nerve roots of L4, L5, S1, S2, and S3; from here, it leaves the pelvis through the greater sciatic foramen and descends on the back of the leg, innervating the posterior region of the thigh, the lower leg, and the foot. It comes out of the pelvis inferiorly to the PM; however, different anatomical variations have been described regarding the relationship between the SN and the PM. In addition, the SN usually divides into its two branches in the lower thigh or in the popliteal fossa; however, this division can also occur at the level of the pelvis or at a different region of the thigh. Anatomically, the SN is closely related to the internal twin-obturator complex. This relationship results in a reproducible dynamic behavior of the SN during passive hip rotation, which may contribute to explaining the pathological mechanisms of the internal twin syndrome of the obturator [6]. Magnetic resonance imaging (MRI) is a technique that seems not to have reached its limit yet and, as well as computed tomography, is used to detect nerve inflammation and evaluation of the thickness of the PM. MRI neurography represents an important advancement in recent years, and it is considered a complementary and non-invasive diagnostic tool in the evaluation of nerves and plexuses, allowing the direct visualization of nerve structures; thus, providing greater diagnostic accuracy compared to other study methods [7].

Anatomical variations of the SN related to its relationship to the PM can lead to conditions such as piriformis syndrome, and should be considered to avoid nerve injury when performing procedures such as pelvic surgeries, hip arthroplasty, intramuscular injections, and cannulation of the spine femoral artery in heart surgery [8]. Clearly, understanding the normal anatomy of the human body and its relationships with other organs is of great importance when performing any type of therapeutic or surgical intervention; thus, it is of great importance to understand which kind of anatomical variations can occur in this region and specify the normal distribution of any anatomical element, the variants, and the most frequent relationships in the case of finding one variation. The SN is formed at six weeks of the embryonic stage and by eight weeks, the PM is formed. This suggests that anatomical variations could be generated in stages before the acquisition of definitive muscle insertion, which is evident at around 15 weeks of development [9]. The importance of the abnormal arrangement of the branches of the SN has implications in the possibility of injury during the application of intramuscular injections, causing failures in the anesthetic block of the nerve, injuries during surgeries in the gluteal region, and the development of piriformis syndrome. It is further suggested that when anatomical variants of the sciatic nerve occur, there is a risk of causing injury during hip arthroplasties, both from direct trauma and from stress caused by traction and manipulation during the surgery [9]. Knowing the possible anatomical variations, and the course of the SN and PM becomes important if it is considered that the presentation of one or another type could influence the development of certain pathologies, or increase the risks of nerve injury during therapeutic practices.

The aim of this paper is to examine and identify SN variations in relation to the PM, as also its prevalence, pattern, and course. To do so, 20 anatomical bodies (corpses) were analyzed at the Forensic Pathology Department of the Central Clinical Hospital of the Academy of Sciences of the Russian Federation, Moscow, Russia.

## 2. Materials and Methods

### 2.1. Data Acquisition

A prospective-descriptive cross-sectional study was carried out to determine the frequency of anatomical variations in the exit of the SN in relation with the PM in 20 anatomical bodies (corpses) of both genders, in equal numbers, analyzed at the Forensic Pathology Department of the Central Clinical Hospital of the Academy of Sciences of the Russian Federation, Moscow, Russia, during the period between January 2022 and April 2022.

### 2.2. Dissection Technique

Data collection was carried out through the dissection of 40 SNs in the corpses of both sexes in equal numbers from the Forensic Pathology Department of the Central Clinical Hospital of the Academy of Sciences of the Russian Federation, under the technique’s dissection of this region. Inclusion criteria are that the corpses had the gluteal region in optimal condition and well-preserved to allow the dissection and data collection. The data collection began with the purpose of processing this information, which was obtained and collected in a previously prepared and validated form. The data were tabulated and graphed in Microsoft Word and Microsoft Excel in the process of writing the results, conclusions, and recommendations of this study.

### 2.3. Ethical and Bioethical Principles

To carry out this research, the principle of confidentiality was preserved in order to protect all the information provided, which was used solely for scientific purposes. Likewise, the norms provided by the institution and other ethical principles, such as justice, beneficence, autonomy, and non-maleficence, were respected.

## 3. Results

**Figure 1:**
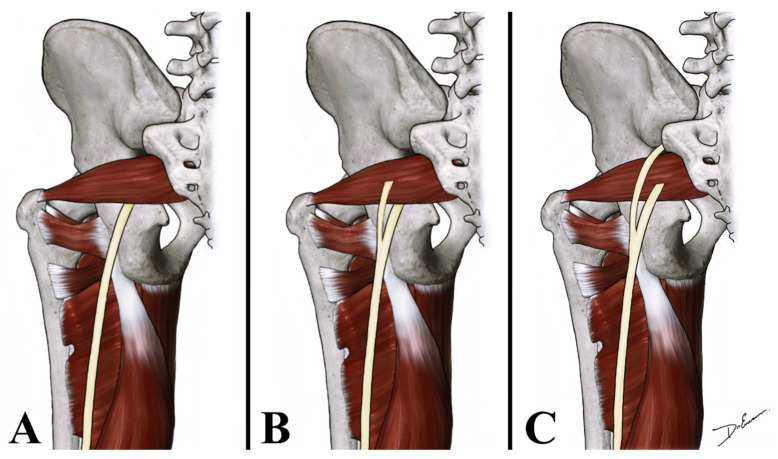
Anatomical drawing shows the relationship of the SN with the PM. SN can exit from the pelvis inferior to the PM (**A**); between the fascicles of the PM and inferior to the PM (**B**); and between the fascicles of the PM and superior to the PM (**C**).

**Figure 2:**
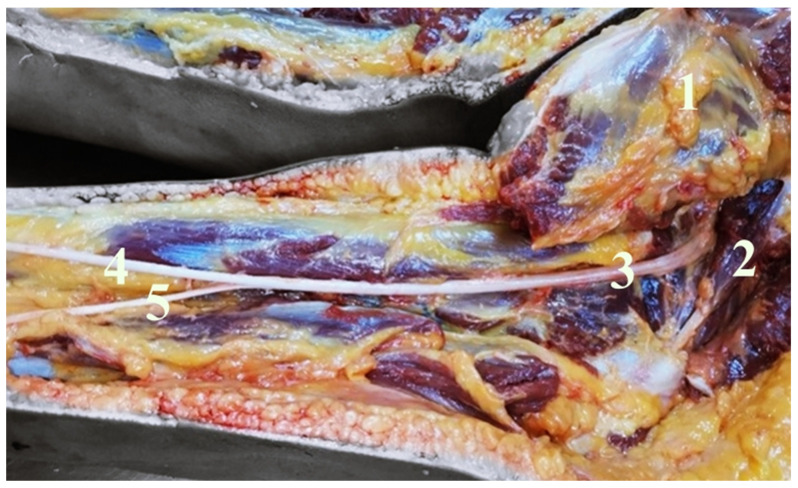
It shows a right deep gluteal and right posterior femoral region. After gluteal major muscle retraction (1), the PM (2) and the SN (3) are visible. The SN exit inferior to the PM, dividing into the tibial nerve (4) and the common peroneal nerve (5).

**Figure 3:**
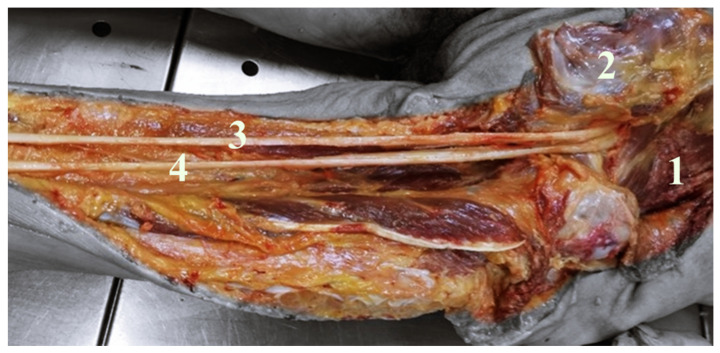
It shows a deep right gluteal region and right posterior femoral region. Gluteal major muscle retracted (1), PM (2). In this case, the SN divides into tibial nerve (3) and common peroneal nerve (4) in the gluteal region.

**Figure 4:**
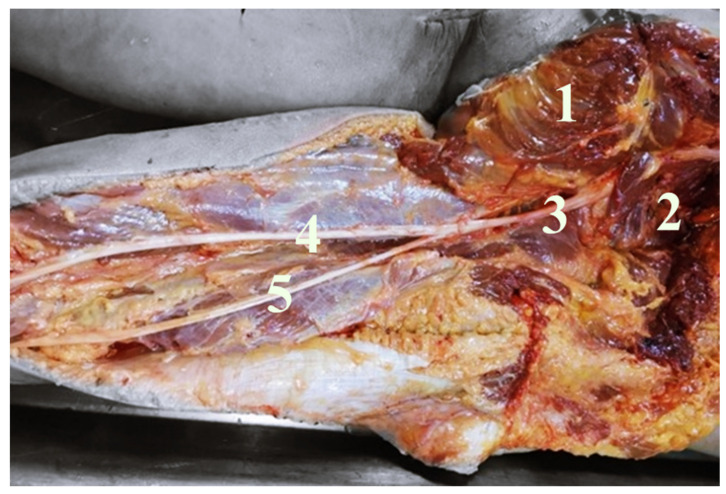
It shows a deep right gluteal region and right posterior femoral region. Gluteal major muscle retracted (1), PM (2). In this case, the SN (3), passing inferior to the PM, divides into tibial nerve (4) and common peroneal nerve (5) in the middle third of the thigh. PM dividing into the posterior femoral region upper third in its terminal branches, (4) tibial nerve, and (5) common peroneal nerve.

In order to examine and identify SN variations in relation to the PM, its prevalence, pattern, and course, the dissection of 40 SNs in corpses of both sexes in equal numbers were analyzed. In our study the SN exited inferior to the PM in 37 lower limbs (92.5%); between the fascicles of the PM and inferior to the PM in two lower limbs (5%); and in one thigh, between the fascicles of the PM and superior to the PM (2.5%) (Figure 1). The anatomical variations that are always unilateral occurred more frequently in the left side (10%) compared to the right side (5%) and were more frequent in female (10%) than in male (5%); however, objectively, these data lack of statistical significance due to the small sample of corpses. Our study reported that the SN divides in its terminal branches more commonly in the proximal part of the popliteal fossa in 55% of cases (Figure 2), in the gluteal region in 35% of cases (Figure 3), and in the middle third of the thigh in 10% of cases (Figure 4). Table 1 shows all the details.

## 4. Discussion

Peripheral neuropathies constitute an important cause of morbidity, with great economic and labor impact. To consider this impact, entrapment neuropathies, the most numerous, generate approximately 100,000 surgical procedures annually in the US and Europe [10]. The diagnosis is fundamentally clinical and can be complicated since the symptoms are imprecise and can be confused with other pathologies. It is estimated that at least 6% of patients diagnosed with low back pain suffer from piriformis syndrome [11].

In 1912, Testut [12] made the first description of the possible anatomical variations between SN and PM. He reported the premature bifurcation of the SN and established four distinct provisions, including the passage of one of the branches of the nerve in a position superior to the PM. Beaton and Anson [13] classified variations of the PM and SN in 120 specimens in 1937, and in 240 specimens in 1938 [14]. Their classification, known as the Beaton and Anson classification [13], was as follows: Type 1 (undivided nerve below undivided muscle), Type 2 (divisions of nerve between and below undivided muscle), Type 3 (divisions above and below undivided muscle), Type 4 (undivided nerve between heads), Type 5 (divisions between and above heads), and Type 6 (undivided nerve above undivided muscle). Calvo et al. [9] conducted a literature review where different variations concerning the SN and PM were established. According to Pooja et al. [15], the knowledge of the normal anatomy of the emergence of the SN from the pelvic cavity after its formation, and the possible variations in relationship with the PM helps in the management and approach of the area in surgical procedures that involve the gluteal region and the SN territory. Güleç et al. [16] reported four clinical cases with piriformis syndrome in which diagnostic ultrasound evaluation of the gluteal region for each patient revealed anatomical variations of the SN, suggesting that in the case of anatomical variations of the SN and PM, the use of ultrasound could increase the accuracy of injection and surgical procedures and reduce their complications.

In our study, the SN exited inferior to the PM in 37 lower limbs (92.5%), and anatomical variations occur in 7.5% of cases. To detect the variable relationship between SN and PM, Berihu et al. [17], dissecting 56 lower limbs, reported that 75% of lower limbs showed normal anatomy of SN, whereas 25% of cases showed variations in relation to PM with trifurcation of the SN in 5% of cases. Monte De Oca [18] reported that the frequency of anatomical variations in the exit of the SN in relation with the PM was 10%, and the most common level at which the SN divided in the terminal branches was at proximal part of the popliteal fossa (75%). Budhiraja et al. [19] conducted a study on 60 lower limbs, reporting a 31.7% of anatomical variations of the SN in relationship with the PM. In particular, the SN emerged between and below the undivided PM in 13.3% of cases, and the common peroneal nerve emerged above the PM with the tibial nerve that emerged below the PM in 18.3% of cases [19].

Similarly, Atoni et al. [20], analyzing 56 lower limbs, reported that 92.9% of cases showed normal anatomy of the SN, whereas four cases (7.1%) showed variations in the morphology of the SN. Natsis and colleagues [21] reported the biggest series, with 294 lower limbs. According with their paper, the SN and PM relationship followed the typical anatomical pattern in 275 limbs (93.6%), whereas in 4.1% of cases, the common peroneal nerve passed through and the tibial nerve below the PM; in one case (0.3%), the common peroneal nerve coursed superior and the tibial nerve below the PM; in one case (0.3%), both nerves penetrated the PM; in one case (0.3%), both nerves passed above the PM and in four cases (1.4%), presented non-classified anatomical variations [21]. Ogeng’o et al. [22] investigated variations of the SN in 82 cadavers of black Kenyans, reporting that in 20.1% of cases, division of the SN occurred in the pelvis, whereas in 79.9% of cases, division occurred outside the pelvis with a single trunk SN exited below the PM.

Barbosa et al. [23] conducted a systematic review and showed that the most prevalent anatomical variation was that the common fibular nerve passed through the piriformis muscle fibers (33.3%) and pointed to a possible association of this condition with piriformis syndrome. Similarly, Poutoglidou and colleagues [24] wrote a comprehensive systematic review with a meta-analysis of the SN variants relative to the PM and compared those variants’ prevalence among different geographical populations with respect to gender and laterality, reporting that SN variants were more common among East Asians (with a 31% pooled prevalence of total variants), and no statistically significant differences with respect to gender and laterality.

All these variations should be considered during the semiology of disorders involving parts of the lower limbs. Given these differences, we believe that large-scale research should be carried out in a bigger multiethnic population group to confirm the associations of this anatomical variation in relation with the PM, which would also provide more information on the frequency of the variations. Although, in recent years, the development of new neurosurgical techniques and 3D devices have helped surgeons to improve their knowledge of surgical anatomy, real laboratory anatomical dissections are needed to safely perform surgeries [25,26,27,28]. The exact position of the SN during surgical procedures around the hip and the variability that has been described may reduce the risk of iatrogenic injury. As the position of the SN is highly variable in its course and bifurcation, ultrasound should be used to identify the position of the nerve and its bifurcation point prior to nerve blocks. The use of ultrasound may increase the success rate, and reduce complications associated with sciatic or popliteal blocks.

### Limitations of the Study

The main limitation of this study is the small sample of corpses. Another limitation is that our anatomical study reflects a single-center experience; therefore, all dissection were carried out using the same technique, with a very small probability that some anatomical variations were missed. This can create some problems in the quality and risk of bias assessment; however, this bias was minimized as the 20 anatomical bodies that met the inclusion criteria were sequentially analyzed. Additional prospective studies should be conducted in an international, multi-center setting, with a large sample size to assess the presence and incidence of these anatomical variations in the SN.

## 5. Conclusions

Anatomical variations of the SN in relation to the PM are challenging for the diagnostic and therapeutic procedure in many clinical and surgical cases. Rapid recognition of the SN changes makes surgical approaches more accurate and effective. Our study confirmed that the SN exits the pelvis most commonly below the PM (92.5%), although some anatomical variations may occur (7.5%); and that the SN divides in its terminal branches more commonly in the proximal part of the popliteal fossa (55%) as well as in the gluteal region (35%). Emphasizing the possible anatomical variations of the SN nerve, and the importance that this has for the different clinical and surgical procedures that are related to the SN can contribute their knowledge to research for the developing application of health science. In the description of the sacral plexus during teaching, the possible anatomical variations that the SN presents in its origin, path, and terminal branches should be explained. Further multicentric studies with great numbers are needed.

## Figures and Tables

**Table 1:** Anatomical variations of the SN in relation to the PM and course.

Characteristics	Number	(%)
Overall anatomical bodies	20	100
Sciatic nerve studied	40	100
Sex		
Male	10	50
Female	10	50
Anatomical variations of the SN exit from the pelvis		
Inferior to the PM	37	92.5
Between the fascicles of the PM and inferior to the PM	2	5
Between the fascicles of the PM and superior to the PM	1	2.5
Anatomical variations of the SN exit from the pelvis among sex		
Male	1/20	5
Female	2/20	10
Anatomical variation of the SN according to the side of the body		
Unilateral	3/3	100
Bilateral	0/3	0
Side of anatomical variation of the SN		
Right	1/20	5
Left	2/20	10
Region of division of the SN into tibial nerve and the common peroneal trunk		
Gluteal region	14	35
Middle third of the thigh	4	10
Proximal part of the popliteal fossa	22	55

## Data Availability

Not applicable.
